# Reversibility and Low Commitment to Forward Catalysis in the Conjugation of Lipid Alkenals by Glutathione Transferase A4-4

**DOI:** 10.3390/biom13020329

**Published:** 2023-02-09

**Authors:** Michele Scian, Lorela Paço, Taylor A. Murphree, Laura M. Shireman, William M. Atkins

**Affiliations:** Department of Medicinal Chemistry, University of Washington, Seattle, WA 98195-7610, USA

**Keywords:** enzyme detoxication, substrate promiscuity, hydroxynonenal, lipid peroxidation, lipid alkenals, enzyme mechanism, deuterium exchange

## Abstract

High concentrations of electrophilic lipid alkenals formed during oxidative stress are implicated in cytotoxicity and disease. However, low concentrations of alkenals are required to induce antioxidative stress responses. An established clearance pathway for lipid alkenals includes conjugation to glutathione (GSH) via Michael addition, which is catalyzed mainly by glutathione transferase isoform A4 (GSTA4-4). Based on the ability of GSTs to catalyze hydrolysis or *retro*-Michael addition of GSH conjugates, and the antioxidant function of low concentrations of lipid alkenals, we hypothesize that GSTA4-4 contributes a homeostatic role in lipid metabolism. Enzymatic kinetic parameters for *retro*-Michael addition with trans-2-Nonenal (NE) reveal the chemical competence of GSTA4-4 in this putative role. The forward GSTA4-4-catalyzed Michael addition occurs with the rapid exchange of the C2 proton of NE in D_2_O as observed by NMR. The isotope exchange was completely dependent on the presence of GSH. The overall commitment to catalysis, or the ratio of first order k_cat,f_ for ‘forward’ Michael addition to the first order k_cat,ex_ for H/D exchange is remarkably low, approximately 3:1. This behavior is consistent with the possibility that GSTA4-4 is a regulatory enzyme that contributes to steady-state levels of lipid alkenals, rather than a strict ‘one way’ detoxication enzyme.

## 1. Introduction

The cytosolic glutathione transferase A4 (GSTA4-4), first characterized in detail by Mannervik and colleagues [1] and Board et al. [2], contributes to the clearance of toxic lipid alkenals formed during oxidative stress. Alkenals of particular interest include 4-hydroxynonenal (HNE), which is a major product of arachidonic acid oxidation, and trans-2-Nonenal (NE) which is derived from the oxidation of omega-7 unsaturated fatty acids. High concentrations of HNE are associated with Alzheimer’s disease, Parkinson’s disease, aging, diabetes, cardiovascular disease, ferroptosis and other diseases [3,4,5,6,7,8]. The lipid peroxidation product NE is a constituent of body odor that increases with aging [9,10].

**Figure 1:**
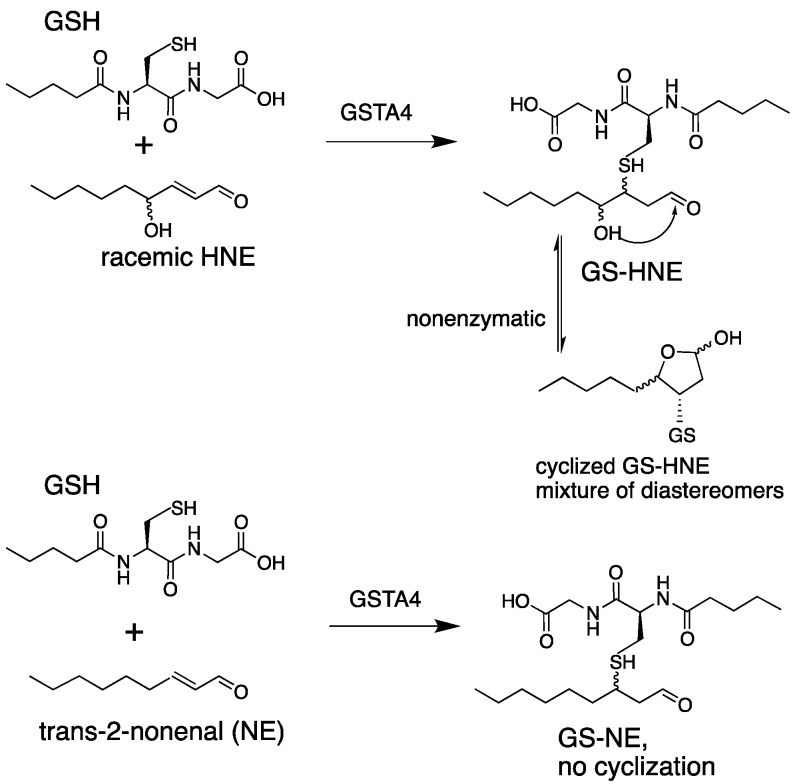
GSTA4-4-catalyzed reaction of lipid alkenals with GSH. The GS-HNE product (**top**) undergoes nonenzymatic cyclization to a mixture of isomers. The GS-NE product (**bottom**) does not cyclize. Curved lines indicate stereochemical heterogeneity.

Many studies indicate that lipid alkenals are not solely electrophilic toxins. At low concentrations, lipid alkenals are intracellular ‘second messengers’ that contribute to homeostasis by managing the expression of antioxidant enzymes [11,12,13,14,15,16]. Among the canonical glutathione transferases (GSTs), GSTA4-4 is highly efficient at catalyzing the ‘Michael addition’ (or ‘conjugate addition’) of lipid alkenals to GSH (Figure 1), and it contributes to their clearance in vivo [17,18]. In comparison, long-chain lipid alkenals are metabolized very slowly by the highly homologous GSTA1-1. GSTA1-1 is extraordinarily substrate promiscuous and is an ‘archetypal’ detoxication enzyme, but with little activity toward lipid alkenals.

**Figure 2:**
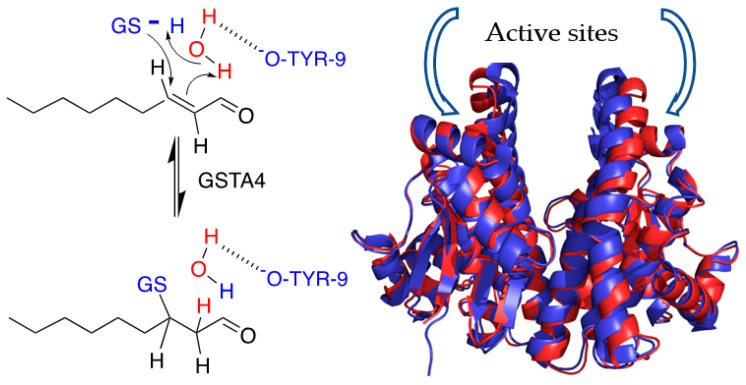
(**Left**): Proposed mechanism for GSH reaction with NE. The active site Tyrosine-9 (Tyr-9) is acidic and remains unprotonated. GSH is initially protonated in contrast to other GSTA isoforms. (**Right**): Superimposition of ligand-free GSTA4-4 (red, PDB1GUL) and GSTA1-1 (blue, PDB1K3L). Each monomer has an active site.

This striking contrast of GSTA4-4 vs. the substrate promiscuity of GSTA1-1 has been studied in detail and quantified [19,20,21,22,23,24]. Structural and thermodynamic contributions to the differences in substrate specificity have been identified. Based on the comparison of the subtle differences in the static crystal structures, Mannervik et al. have identified key structural determinants of the specificity of GSTA4-4 and incorporated them into GSTA1-1 to elegantly redesign its activity to metabolize NE [20,21]. Although the two GST isoforms are nearly superimposable in static crystal structures (Figure 2) of the ligand-free enzymes, critical differences in the global dynamics of the proteins and of the C-terminal helix (residues 209–222) have been described [20,22,23,24]. GSTA4-4 and the redesigned GSTA1-1 are rigid templates with an immobile, ligand-insensitive, C-terminal helix that contributes one side of a long active site [20,25]. In contrast, the C-terminus of GSTA1-1 is highly mobile and adopts ligand-dependent conformations upon binding [20,26,27,28]. The C-terminus of GSTA1-1 is a mobile ‘lid’ over the active site. In addition, the C-terminus of GSTA1-1 uniquely exhibits thermodynamic heterogeneity, or ‘molten globule’ behavior, and its conformational flexibility likely contributes to its substrate promiscuity [29,30]. Structural and dynamic contributions to the substrate selectivity vs. promiscuity of these GSTs are well characterized.

In addition to differences in protein dynamics, the ionization behaviors of active site residues also distinguish the two isoforms. The conserved active site Tyr-9 is mostly protonated at pH 7.4 and hydrogen bonded to the GS^-^ thiolate in GSTA1-1 [31,32]. The pK_a_ of Tyr-9 is tuned by active site features to optimize this hydrogen bond [33,34]. In contrast, the highly acidic Tyr-9 of GSTA4-4 is unprotonated and the tyrosinate likely forms hydrogen bonds to a water molecule in GSTA4-4 [20,31], and the same water hydrogen bonds to the protonated GSH. Thus, the protonation states of GSH and Tyr-9 are opposite in GSTA1-1 vs. GSTA4-4. Mannervik et al. have proposed a detailed mechanism for GSTA4-4-dependent Michael addition of GSH to alkenals, based on these considerations [31] (Figure 2).

The lack of substrate-dependent conformational change with GSTA4-4 is enigmatic because conformational changes are commonly used by enzymes to achieve substrate specificity [35]. Conformational change after the substrate binding, or ‘induced fit,’ can increase the flux of substrate to the product by decreasing substrate dissociation and increasing the commitment to catalysis, which is defined qualitatively here as the tendency of enzyme-bound intermediates to partition forward toward the product vs. back to the substrate [35]. Similarly, rapid conformational change after product formation can minimize backward conversion to substrate and facilitate the release of the product to ensure a high commitment to catalysis. In the absence of conformational changes, enzymes do not optimize their directional commitment to catalysis. Based on the apparent lack of conformational change when GSTA4-4 binds ligands, we explored its reversibility with alkenal substrates. Although GSTA4-4 has been reported to catalyze the *retro*-Michael addition with GS-HNE at detectable rates, a detailed kinetic profile has not been reported [36]. Here, we studied the *retro*-Michael addition of GS-NE to understand the enigmatic behavior of GSTA4-4 compared to GSTA1-1. This detailed analysis indicates that GSTA4-4 is relatively inefficient in the forward direction in as much as it has a low commitment to catalysis, despite having high substrate specificity. To the extent that enzyme parameters are tuned by evolutionary pressure for specific functions, the results prompt speculation of a possible role for GSTA4-4 in regenerating lipid alkenals and GSH during extreme oxidative stress, in contrast to its presumed role as a ‘one way’ detoxication enzyme.

## 2. Materials and Methods

### 2.1. Protein Expression and Purification

Human GSTA4-4 and GSTA1-1 were expressed in *E. coli* and purified by affinity chromatography using S-hexylglutathione Sepharose as previously described [37,38]. The purity of the proteins was confirmed by SDS-PAGE. The purified protein solutions were then dialyzed against 100 mM sodium phosphate buffer (NaPi), pH 6.5, to remove GSH. Stock solutions of freshly prepared enzymes were finally buffer exchanged with 100 mM NaPi, pH 6.5, in D_2_O and aliquots were stored at −80 °C. To ensure reproducibility, all the reported NMR experiments were conducted on aliquots of the same protein preparations. The following molar extinction coefficients were used to determine the folded protein dimer concentration: GSTA1-1, e_280_ = 46,147 M^−1^cm^−1^; GSTA4-4, e_280_ = 34,300 M^−1^cm^−1^. All enzyme concentrations are reported as concentrations of dimeric species.

### 2.2. UV-Vis Assays for Forward and Reverse Reactions

Reaction velocities for GSTA4-4- and GSTA1-1-catalyzed GSH conjugation to NE were determined in comparable conditions to the ones used in the H/D exchange NMR kinetic assay by monitoring depletion of NE at 224 nm via UV spectroscopy using the extinction coefficient: ε_224 nm_ = 19,220 M^−1^cm^−1^. In the reaction of 20 nM of GSTA4-4 with 20 μM GSH and 200 μM NE in 100 mM KPi, pH 6.76, 2% (*v*/*v*) ethanol was monitored for 1 min at 25 °C and the reaction velocity was calculated as the amount of NE depleted over time in the linear range of the reaction (30 s). In the reaction of 5 nM GSTA1-1 with 40 μM GSH and 200 μM NE in 100 mM KPi, pH 6.5, 1% (*v*/*v*) ethanol was monitored for 1 min and the reaction velocity was calculated as the amount of NE depleted over time in the first 30 s of the reaction. The dead time of the assay (between initiation and measurement was ~10 s. The K_M, GSH_ for GSH (fixed NE, co-substrate), V_max_, and k_cat,f_ kinetic parameters for the forward reaction of GSTA4-4 with NE were determined by monitoring the depletion of NE at varying concentrations of GSH. A mix of 20 nM GSTA4-4 with 200 μΜ NE was treated with 20 μΜ–3 mM of freshly prepared reduced GSH to initiate the reaction in 100 mM KPi, pH 6.7, 5% (*v*/*v*) ethanol at 25 °C. Due to the high extinction coefficient of NE, we were limited to using a subsaturating concentration in the reaction mixture to avoid saturating the UV detector. Reaction velocities calculated from the amount of NE depleted in the linear range (10–30 s) of the reaction were corrected for the contribution of the spontaneous reaction in the range of GSH concentrations used and fit to the Michaelis Menten (MM) model of enzyme kinetics to obtain kinetic parameters in GraphPad Prism. The K_m,GSNE_, V_max_, and k_cat,r_ kinetic parameters for the reverse reaction of GSTA4-4 with NE were determined by monitoring the appearance of NE at 224 nm via UV spectroscopy. GSH, the second product of the reverse reaction, also absorbs at 224 nm but its contribution to absorbance is negligible at the concentrations of GS-NE used. A total of 20 nM of GSTA4-4 was treated with 0.5 μM–200 μM GS-NE in 100 mM KPi, pH 6.7, 5% (*v*/*v*) ethanol at 25 °C. The concentrations of GSTA4-4 and GS-NE used were determined from preliminary experiments to allow for the linear formation of NE in the monitored reaction time (~30 s) and an adequate range of GS-NE concentrations. As in the forward direction, the reaction velocities were corrected for the contribution of the spontaneous reaction, and kinetic parameters were obtained by fitting the data to the MM model in the GraphPad Prism.

### 2.3. GS-NE Synthesis

GS-NE synthesis was initiated by mixing 500 mL of 1 mM NE (97%, Sigma-Aldrich, St. Louis, MO, USA) with 2 mM GSH in 50 mM KPi, pH 7.0, 2% (*v*/*v*) DMSO at RT. The reaction was monitored by ^1^H NMR for 6 h before lowering the pH to 3.0 with 1 M HCl. The reaction mixture was then applied to SPE cartridges (Solid Phase Extraction with Waters Sep-Pak tC18, 6 cc Vac cartridge, 500 mg sorbent) in 25 mL aliquots for bulk separation of reaction mix components. Following the loading of each aliquot, the SPE cartridge was washed with 2 × 5 mL 0.1% FA and 3 × 5 mL H_2_O and the GS-NE product was eluted with 1 mL of 70% ACN. At −20 °C the 70% ACN solution forms two phases, where GS-NE is contained in the bottom aqueous layer. The latter was purified by RP-HPLC with UW detection at 254 nm and a Syncronis aQ, 10 × 250 mm, 5 μm C18 column (Thermo Scientific) using a 5–74% mobile phase B gradient for 11.5 min at 4 mL/min (A: H_2_O + 0.1% FA; B: ACN + 0.1% FA). The isolated LC fractions were analyzed by NMR (1D; 2D COSY, 2D TOCSY) and MS (direct infusion to Waters Synapt G1) to confirm the formation of pure GS-NE product (Supporting Information, Figures S1–S4).

### 2.4. LC-Mass Spectrometry

Samples for GST-catalyzed and spontaneous reactions of NE with GSH in the forward and reverse direction were prepared for preliminary determination of kinetic parameters via LC-MS. In the ‘forward reaction’ samples, 150 or 160 μL of 50 mM KPi, pH 6.5, and 20 μL of 10× reduced GSH solution freshly prepared in the same buffer were mixed with 20 μL of 10× NE stock in 20% ethanol, with or without 10 μL of 20× GST stock solution for a total reaction volume of 200 μL. The final concentration of GSH was kept constant at 1.5 mM for all ‘forward reaction’ samples. A total of 20 nM GSTA4-4 or 1 μΜ GSTA1-1 (Sigma) was used in the GST-catalyzed samples. The final concentration of NE was 0, 20, 50, 100, 200, 500, 750, 1000, or 1500 μM in the GSTA4-4-catalyzed samples and 0, 5, 10, 20, 50, 100, 200, 500, and 750 μM in the GSTA1-1-catalyzed samples. NE concentrations were chosen based on kinetic parameters obtained with both enzymes by the UV assay. In the ‘reverse reaction’ samples, 20 μL of 10× GS-NE stock solution in 20% ethanol was added to 170 or 180 μL buffer with or without 10 μL of 20× GST stock solution. The final concentration of GS-NE was 0, 5, 10, 20, 50, 100, 200, 500, and 750 μM in the GSTA4-4-catalyzed samples, and 0, 5, 10, 20, 50, 100, 200, 500, 750, and 1000 μM in the GSTA1-1-catalyzed samples. Forward and reverse reaction samples were prepared in duplicate in a well-plate format and all were at 25 °C.

### 2.5. NMR Assignment of NE

The concentrations of NE (97%, Sigma-Aldrich, St. Louis, MO, USA) and stock solutions in ethanol-d6 were determined by UV-vis as previously reported (e_225_ = 19,220 M^−1^cm^−1^ and e_224_ = 13,750 M^−1^cm^−1^, respectively). For assignment purposes, NMR spectra were acquired at a resolution of 32 k points in the time domain (16 k complex) and with 64 accumulations each (sw = 6000 Hz, d1 = 3 s), with sodium 2,2-dimethyl-2-silapentane-5-sulfonate (DSS) as the internal chemical shift reference [39].

### 2.6. The GSH-Dependence of Deuterium Exchange

Aliquots of GSTA4-4 stock solutions were diluted to 10 μM with 100 mM deuterated NaPi buffer at pH * = 6.66 (purged with Argon for 1 min), where pH * is the reading on a standard electrode where pH * = pD + 0.4 and pD = −log [D^+^]. The concentrations were rapidly determined by UV-vis. To appropriate amounts of these solutions, freshly made L-Glutathione (≥98%, Sigma-Aldrich, St. Louis, MO, USA) solutions were added (reduced GSH in 100 mM deuterated NaPi, pH * = 6.66, purged with Argon for 1 min) to a final enzyme concentration of 10 nM and [GSH] = 1.5, 2.5, 5.0, 10 mM (in 600 mL total). Lastly, NE (27.5 mM stock solution in ethanol-d6) to a final concentration of 275 μM was added, immediately before starting the NMR experiment. The co-solvent concentration in each of the NMR samples was kept to 1% *v*/*v*. The contribution of the residual Glutathione present in the enzyme stock solutions was negligible, due to the low protein concentration used for these experiments. A total of 25 individual spectra (256 scans for [GSH] = 1.5 and 2.5 mM, 128 scans for [GSH] = 5.0 and 10.0 mM, 4 k complex points, sw = 6000 Hz, d1 = 1.0 s) were collected with WATERGATE solvent suppression. The dead time, t_0_, between mixing and acquisition was around 3–5 min and no pre-acquisition delay between experiments was used. A 1 Hz line-broadening apodization function and zero-filling to 8 k complex points were applied before the Fourier transformation. The C2 proton resonance integrals (normalized to 1 at t_0_) were fitted as a function of time to a single exponential decay. All exchange experiments were at 25 °C.

### 2.7. Equilibrium of GSH, NE and GS-NE by NMR

Aliquots of GSTA4-4 or GSTA1-1 (~235 nM) stock solutions were diluted to ~15 μM with 100 mM NaPi buffer, 10% D_2_O, pH * = 6.82 and the concentrations were measured. These solutions were used to prepare the NMR samples (600 mL total) by adding freshly made l-Glutathione stock solutions (reduced GSH in 100 mM NaPi, 10% D_2_O, pH * = 6.82) and trans-2-NE (27.5 mM stock solution in ethanol-d6). The final concentrations were as follows: 10 nM enzyme, 1.0 mM GSH, 275 mM NE 1% *v*/*v* ethanol-d6. The mixtures were allowed to react for one hour while monitored by NMR. At equilibrium, more than 85% of the product was formed. To rapidly oxidize the unreacted GSH, 40 mL of a H_2_O_2_ stock solution ~0.882 M (in 100 mM NaPi buffer, 10% D_2_O, pH * = 6.82) was then added at a final concentration of ~55 mM and the *retro*-Michael addition was monitored by NMR. A total of 10 individual spectra were collected for A4-4 (256 scans, 16 k complex points, sw = 6000 Hz, d1 = 1 s), whereas 30 spectra were collected for A1-1 (256 scans, 16 k complex points, sw = 6000 Hz, d1 = 1 s). WATERGATE solvent suppression and a 30 ms T1_rho_ filter were used. Dead time, t_0_, between mixing and acquisition was around 5–10 min and no pre-acquisition delay between experiments was used. A 1 Hz line-broadening apodization function and zero-filling to 32 k complex points were applied before the Fourier transformation.

### 2.8. Deuterium NMR of NE Recovered from Enzyme Incubation

A 60 mL solution 10 nM GSTA4-4, 0.5 mM NE, 1% *v*/*v* CH_3_CN, in 100 mM NaPi pH * 6.82 in D_2_O were incubated at room temperature for 8 h. The progress of the deuteration reaction was monitored by NMR on a 600 mL aliquot. The mixture was then applied to a centrifugal filter unit (Millipore, 10 kDa MWCO, 50 mL volume) and the filtered was passed, under vacuum, through a disposable C18 column (disposable BAKERBOND Reversed Phase Octadecyl SPE Column, J.T. Baker, 1 mL volume, 100 mg sorbent weight), to immobilize the NE. The C18 column was then washed with 10 × 1 mL H_2_O, dried by centrifugation at 3000× *g* for 10 min and eluted with 350 μL CH_3_CN. The eluted was stored at −80 °C. After the elution, the C18 column was washed with 5 mL CH_3_CN, 5 mL H_2_O and then with 1 mL D_2_O to remove the residual H_2_O for the next immobilization step. The whole incubation/extraction process was repeated after the addition of a fresh aliquot of GSTA4-4 (to a 10 nM final concentration) and NE (to a 1 mM final concentration) at the extracted solution. The eluted fractions (2 × 350 mL) were pooled together, treated with Na_2_SO_4_ for 24 h and transferred into an NMR tube. The ^2^H NMR spectrum (4096 scans, 2048 complex points, sw = 900 Hz, d1 = 3 s) was collected using the lock channel of a triple resonance, inverse detection probe, and without proton decoupling. The deuterium resonance of CD_3_CN (99.96 atom% D, Sigma-Aldrich, St. Louis, MO, USA) was used as internal chemical shift reference and set to 1.94 ppm. A 1 Hz line-broadening apodization function and zero-filling to 4 k complex points were applied before Fourier transformation.

## 3. Results

To characterize in greater detail the steady state behavior and reaction dynamics of GSTA4-4, we exploited NE as a model substrate. Although HNE is more impactful from a toxicological perspective, its participation in the uncatalyzed approach to equilibrium between ring-closed hemiacetal and ring-opened aldehyde (Figure 1) prohibits analysis of the reaction of interest because the nonenzymatic rates of these reactions are conflated with the enzymatic steps. Therefore, to monitor the reaction dynamics of enzyme-bound states, we focused on NE, which does not cyclize, as a model.

### 3.1. Steady State Forward and Reverse Reaction

The GSTA4-4-catalyzed forward reaction was monitored by two methods. A previously established method based on UV absorbance at 224 nm has been described and provides a convenient method at varying concentrations of GSH (20 μM–3 mM). The UV method is limited at high concentrations of NE, for which the absorbance saturates the detector. With this method, for variable [GSH] and 200 mM NE, initial velocities in the linear range were plotted against GSH concentration and fit with the Michaelis-Menten equation to yield K_M,GSH_ = 530 μM, k_cat,f_ = 89 s^−1^, k_cat,f_/K_M_ = 0.17 μΜ^−1^s^−1^ for the forward reaction, where k_cat,f_ and k_cat,r_ refer to forward and reverse reactions. These parameters are in agreement with previously reported values [1]. As noted above, the concentration of NE was not saturating due to high absorbance at 224 nm, so the k_cat,f_ values are expected to be modestly lower than the true value.

**Figure 3:**
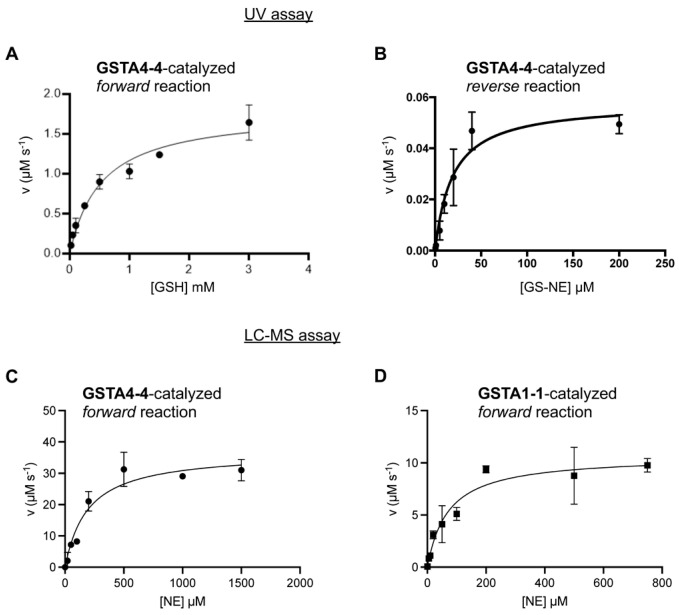
Steady state forward and reverse reactions of GSTA4-4 and forward reaction of GSTA1-1. (**A**): GSTA4-4 activity for the depletion of NE in the ‘forward’ reaction to yield GS-NE, based on the decrease in UV absorbance. (**B**): GSTA4-4 activity in the ‘reverse’ direction to form GSH and NE from GS-NE based on the increase in UV absorbance. (**C**): GSTA4-4 activity in the forward reaction based on LC-MS. (**D**): GSTA1-1 activity in the forward reaction based on LC-MS.

To circumvent the limitations of the UV assay with high concentrations of NE, GS-NE formation was also measured by LC-MS with 20 nM GSTA4-4, 1.5 mM GSH, and varying concentrations of NE (20 μΜ–1.5 mM) in 100 mM KPi, pH 6.5 (Figure 3), resulting in the following kinetic parameters: K_M,NE_ = 195 μM, k_cat,f_ = 184 s^−1^, k_cat,f_/K_M,NE_ = 0.95 μΜ^−1^s^−1^. As expected, the k_cat,f_ value from the LC-MS assay with saturating co-substrate is modestly higher than the k_cat,f_ from the UV assay with non-saturating co-substrate. The data are summarized in Figure 3. Recovered parameters are in Table 1 and they include a low standard error for the k_cat_ values and modest error in the K_M_ values. The error in K_M,GSH_ is high for unknown reasons but the recovered value is in line with well-accepted values in the literature.

For comparison, kinetic parameters for the GSTA1-1-catalyzed reaction of GSH with NE were obtained by LC-MS in the forward direction with 1 μM GSTA1-1, 1.5 mM GSH and varying concentration of NE (5–750 μM) (Figure 3), resulting in K_M,NE_ = 71 μM, k_cat_ = 0.3 s^−1^, k_cat_/K_M_ = 0.0042 s^−1^μΜ^−1^. As expected, based on previous results, GSTA1-1 is much less efficient than GSTA4-4 in conjugating NE with GSH.

For the reverse reaction, synthetic GS-NE was prepared and incubated at varying concentrations with GSTA4-4, as described in Methods. For the reverse reaction, the UV assay works well at a low extent of turnover, prior to the generation of high concentrations of NE. The appearance of NE was measured by increased absorbance at 224 nm with 10 nM GSTA4-4 and varying concentrations of GS-NE (0.5–200 μM) in 100 mM KPi, pH 6.7 (Figure 3). It is important to note that we used a mixture of GS-NE diastereomers. For the mixture, the experimentally determined K_M,GS-NE_ was 19 μM and the k_cat,r_ was 1.2 s^−1^. The kinetic parameters for the reverse reaction are summarized in Table 1. A striking feature of the reverse reaction with GS-NE is the low K_M_. To the extent that K_M_ can be a surrogate for K_D_ in some cases, the affinity of GS-NE for GSTA4-4 is much greater than the substrates for the forward reaction, GSH and NE. Interestingly, we observe no product inhibition in the reverse direction, despite the high affinity for GS-NE compared to GSH or NE. In turn, the lack of product inhibition combined with the relatively high reverse rate suggests the possibility of rapid conversion of bound GS-NE to GSH and NE. For the reverse reaction, GSTA1-1 exhibited no detectable reaction at 10 nM enzyme, which was the concentration of GSTA4-4 used for the reverse reaction.

### 3.2. Solvent Exchange with NE by NMR

**Figure 4:**
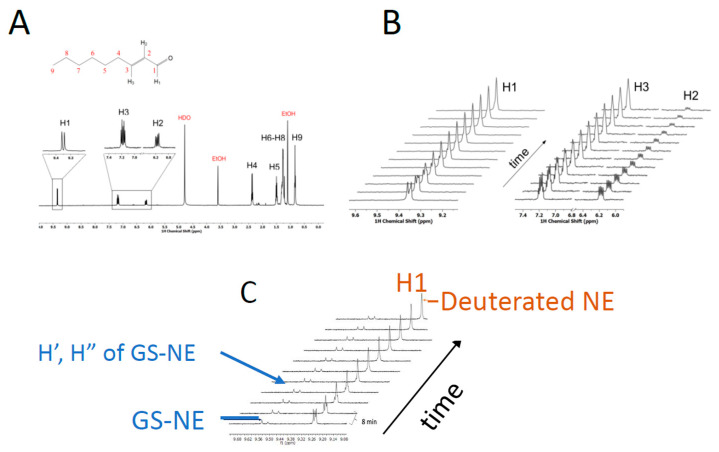
Chemical Structure and ^1^H-NMR spectrum of NE. (**A**) The structure of NE is shown with carbons or corresponding protons numbered. The ^1^H-NMR spectrum of NE is shown below its chemical structure. The H1, H2, and H3 protons provide probes of the D_2_O exchange at carbon 2, or C2. (**B**) Time-dependent changes in H1, H2, H3 upon incubation with GSTA4-4 in the presence of GSH. (**C**) Time-dependent exchange of ^1^H of NE to yield deuterated substrate without change in [GS-NE] after the dead time of 4–5 min. The protons H′ and H″ are from GS-NE formed at low concentrations due to limiting [GSH]. For (**B**,**C**), the arrows show the time dependence of the spectra.

Based on our observation and previous literature reports that GSTA4-4 catalyzes the *retro*-Michael addition of GSH with NE, we considered GST-catalyzed solvent deuterium incorporation into NE as a probe of the relative rates of forward and reverse partitioning of an enzyme-bound Michael adduct. The ^1^H NMR spectrum of NE is shown in Figure 4. When NE was incubated with 10–50 μM GSH and 10 nM GSTA4-4 in D_2_O the NMR signal from the C2 proton of NE rapidly decreased. At the same time, the C3 proton changed from a well-resolved doublet of triplets to a poorly resolved multiplet, without any loss in total peak area. Similarly, the aldehydic proton at C1 changed from a doublet to a singlet without the loss of peak area. Changes in the NMR spectrum occurred before any detectable depletion of NE or without detectable formation of GS-adducts in the NMR spectrum indicating that solvent exchange happens on a time scale that is not significantly slower than product, GS-alkenal, formation. These spectral changes did not occur at any detectable level in the absence of GSTA4-4, on the time scale of these experiments. These results are shown in Figure 4 and they suggest that deuterium was incorporated at C2 of the starting substrate NE in an enzyme-mediated process. The results suggest that an initially formed GS-NE adduct with deuterium incorporated from solvent can readily undergo *retro* Michael addition to yield deuterated NE substrate. For comparison, parallel experiments with GSTA1-1 demonstrated no deuterium into NE except at much longer times and in the presence of 1000-fold more enzyme, as shown in Section 3.6.

A critical observation of the data in Figure 4 is the change in peak splitting of H1 and H3 due to ^2^H-incorporation at C2 without loss of total intensity of H1 or H3, where ^2^H designates deuterium vs. a proton, ^1^H. This suggests either the system is at chemical equilibrium prior to reaching isotopic equilibrium, or isotope exchange is much faster than GS-NE production. In fact, under limiting [GSH] conditions, GS-NE is detectable, albeit with low intensity, and the peak areas of the GS-NE protons were monitored. The H1 proton of GS-NE is shifted to the two diastereotopic singlets H′, H″ at 9.56 ppm and 9.58 ppm upon conjugation to GSH (Figure 4C and Supporting Information Figure S1). Based on the NMR, [GS-NE] does not change during the ^1^H-exchange of NE observed by NMR (5 min dead time). The solvent exchange is not faster than the forward reaction to yield GS-NE. The NMR results report on solvent isotope exchange at equilibrium, as discussed below in Section 3.6.

### 3.3. The Site of Deuterium Incorporation from Solvent Exchange

The observed changes in the ^1^H-NMR spectrum of NE are consistent with the incorporation of deuterium (^2^H) at C2. To demonstrate directly that the ^1^H NMR spectral changes were due to the incorporation of deuterium at C2, NE was isolated from incubations with NE, GSH and GSTA4-4 in D_2_O buffer and its deuterium NMR spectrum was acquired (Figure S5, Supporting Information). The deuterium spectrum unambiguously confirms that solvent deuterons are incorporated at the C2 position and only at the C2 position. No other protons of NE were exchanged with deuterons.

### 3.4. Solvent Exchange Requires GSH

To investigate the mechanism of this exchange reaction we measured the rate of exchange at variable [GSH]. Specifically, we aimed to determine whether an initially formed GS-alkenal conjugate was reversibly collapsing into substrates or whether the reversible addition–elimination of water to the alkenal in the active site was the source of deuterium incorporation. When the rate of solvent deuterium incorporation into NE was determined at variable [GSH], there was a dramatic increase in the rates of exchange with increasing [GSH] up to 20 μM (Supporting Information, Figure S6). Above 20 μM [GSH] the rate of deuterium incorporation became too fast to measure by NMR. Notably, in the absence of added GSH, no exchange was detected over the course of 2 h. The exchange of solvent deuterium at C2 of NE was completely dependent on the presence of GSH and GSTA4-4 under the time scales studied here. The obligate presence of GSH in the exchange reaction indicates that the exchange occurs from an intermediate that lies on the reaction coordinate for the formation of GS-alkenal, presumably the [GST•GS-alkenal] intermediate.

### 3.5. Equilibrium Constant for the Reaction

With the NMR spectra of synthetic GS-NE in hand, the NMR provides a convenient and direct measurement of K_eq_ for the reaction of GSH + NE ⇔ GS-NE. The protons at C1 in the GS-NE (H1′, H1″) are shifted to 9.56 ppm–9.58 ppm and are completely resolved from the H1 of NE. These peaks are assigned as diastereomeric. With 1 mM NE and 2.5 mM GSH the time-dependent conversion to the equilibrium mixture of GSH, NE, and GS-NE was followed. At equilibrium, the ratio of peak integrals for the H1′ and H″ protons in GS-NE to the peak integral for H1 of NE is:

(1) $$\frac{{H1\text{′}}_{\;\mathrm{GS}-\mathrm{NE}}{}_{\mathrm{eq}}}{{H1}_{\;\mathrm{NE}}{}_{\mathrm{eq}}}=\frac{{\left[\mathrm{GS}-\mathrm{NE}\right]}_{\mathrm{eq}}}{{\left[\mathrm{NE}\right]}_{\mathrm{eq}}\;}=16.7\;\text{±}\;5$$

Based on the 1:1 NE:GSH stoichiometric requirement of the reaction, the K_eq_ = [GS-NE]/[GSH][NE] is calculated to be 10.7 mM^−1^. With Keq = 10.7, at equilibrium, approximately 94.4% of the material is product GS-NE and only 5.6% is GSH or NE on a molar basis. The propagated error of the measurement is large in terms of the value of K_eq_ but small in terms of the percentages. For example, with a value of [GS-NE]_eq_/[NE]_eq_ = 11.7 as the lower limit within the standard error for the experimentally measured ratio, instead of 16.7, the distribution of material is 92.2% GS-NE and 7.8% GSH or NE. The K_eq_ measured by NMR indicates the reaction is highly favored in the forward conjugate addition direction and is thermodynamically highly unfavorable in the reverse elimination direction. The reaction to generate GSH and NE from GS-NE is thermodynamically ‘uphill’.

### 3.6. Estimating Relative Rates of Deuterium Exchange into NE and Formation of GS-NE

**Figure 5:**
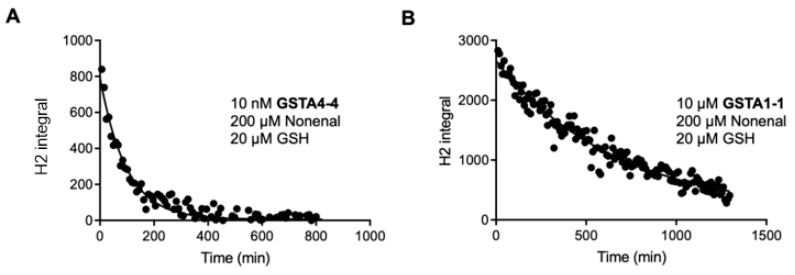
Exponential fits of ^1^H-NE depletion with GSTA4-4 (**A**) or GSTA1-1 (**B**).

The results in Figure 4 demonstrate that the solvent isotope exchange at equilibrium is easily observable. Therefore, to compare the partitioning of the intermediate [GST•GS-NE] complex backwards to form GSH and NE vs. forward to yield GS-NE, we employed a substrate depletion analysis of the ^1^H-NE (undeuterated NE) under equilibrium conditions using the H1 integrated peak area for the proton at C2. Under these conditions, total NE is not changing but the isotopic composition is changing. The time-dependent decrease in integrated peak area of the C2 NMR peak when the reaction was run in D_2_O was fit to a single exponential which yields an empirical first-order rate of ^1^H-NE depletion or solvent exchange, k_dep_, which is a fraction of the true k_cat,ex_ for the exchange because the enzyme is not saturated with GSH or NE under conditions that allow for observation by NMR. The exponential behavior and k_dep_ are validated surrogates for the first-order k_cat_ as long as substrate binding is not rate limiting [40]. At 200 μM NE, 20 μM GSH, and 10 nM GST the half-life for NE deuteration was 66.8 min (Figure 5), which corresponds to a k_dep_ for NE of 149.7/min. When normalized to enzyme concentration and saturating [GSH], the k_cat,ex_ for GSTA4-4-dependent deuteration of NE is 69 s^−1^, which is not much slower than the measured k_cat,f_ for GS-NE formation of 184 s^−1^ determined by the LC-MS assay and even closer to the value determined by the UV assay (Table 1). For comparison, the time-dependent exponential loss of ^1^H-NE was also determined with GSTA1-1 at 100-fold higher enzyme concentration. The results for both GSTA4-4 and GSTA1-1 are shown in Figure 5 and the recovered rates are summarized in Table 2. Sources of potential error in these initial estimates are described in the Discussion. It is notable, however, that GSTA4-4 exhibits a relatively low ratio of forward partitioning compared to backward exchange compared to the much slower GSTA1-1.

Concentrations of reagents are shown in each panel. The integrated peak area of the C2 proton (H2) was fit to a single exponential decay. GSTA1-1 exhibits a detectable exchange but is much slower than GSTA4-4.

## 4. Discussion

The chemical competence of GSTs to catalyze the thermodynamically favored hydrolysis of thioesters or the breakdown of GSH-conjugates has been documented previously [41,42,43,44,45] The mechanistic details of such reverse GST reactions have been explored in a few cases with in vitro chemical probes [41,43]. The potential biological or toxicological effects of toxins delivered to tissues as a result of the breakdown of GSH-conjugates have been demonstrated and discussed [46,47,48]. For example, the release of methyl isocyanate from its GSH conjugate contributed to the toxicological impact of the ‘Bhopal disaster.’ In that case, the GSH conjugate provided a vehicle for the transport of methyl isocyanate throughout multiple organs or tissues [49]. In most cases, the nonenzymatic or GST-mediated reversibility of GSH conjugate formation is likely to be deleterious to the organism.

In contrast, the potential advantages of the reversibility of GSTs have not been considered in detail, including the case of the endogenously formed lipid alkenals. The role of GSTA4-4 in regulating HNE levels in cells has been documented based on the ‘forward’ conjugation to GS-HNE [50,51]. Transfection of cells with GSTA4-4 alters cellular proliferation and oxidative stress responses due to the depletion of HNE. The depletion of HNE apparently leads to altered expression of many other genes related to oxidative stress or cell growth [14,50,51]. Here, we explored the possible contribution of the reversibility of GSTA4-4-catalyzed lipid alkenal conjugation. We hypothesized that GSTA4-4 kinetic behavior could also contribute to the homeostasis of oxidative stress responses by maintaining low concentrations of the lipid alkenals that could possibly activate the NRf2-Keap pathway and by contributing to the regeneration of GSH during oxidative stress. The studies summarized here demonstrate that GSTA4-4 is not evolutionarily optimized as a ‘one way’ detoxication enzyme to clear lipid alkenals. Instead, enzymatic parameters indicate that GSTA4-4 is an efficient catalyst of *retro*-Michael addition with lipid alkenals and it has a low commitment to catalysis in the forward direction to form GSH adducts. Specifically, the k_cat,f_/K_M,NE_ and k_cat,f_/K_M,GSH_ values in the forward direction are only 16-fold and three-fold larger than the k_cat,r_/K_M,GS-NE_ in the reverse direction (Table 1). The results from isotope exchange studies demonstrate that GSTA4-4 catalyzes the exchange of solvent deuterium at C2 of NE on time scales that are comparable to the net conversion of GSH and NE to GS-NE. GSTA4-4 is highly reversible due to both a low K_M,GS-NE_ and rapid conversion of bound GS-NE to GSH and NE. The rapid conversion of bound GS-NE to GSH and NE results in a low commitment to catalysis in the forward direction, as defined in Table 2. It is striking that GSTA1-1 actually has a higher relative commitment to catalysis based on this criterion. Although GSTA1-1 is a very poor catalyst for the metabolism of NE (or HNE) with slow flux in either direction, it has a higher relative flux of enzyme-bound intermediate toward GS-NE than to NE and GSH than GSTA4-4 does.

An accurate estimation of the commitment to catalysis based on NMR and steady-state UV-vis or LC-MS is confounded by stereochemical and kinetic considerations. The requirement for D_2_O as a solvent to measure the incorporation of deuterium into NE obviously introduces possible solvent KIEs in both directions. Two types of solvent KIEs could contribute. There could be D_2_O KIEs on the physical binding steps, such as the release or binding of GS-NE, NE and GSH. Effects of D_2_O on ligand binding are usually less than 2. Because GSTA4-4 undergoes a minimal conformational change upon binding, and binding is controlled by diffusive properties, solvent isotope effects on binding and dissociation would be negligible [52,53,54]. There are more likely to be solvent isotope KIEs on the chemical steps in both directions in as much as multiple protons are likely to be involved in bond making or breaking in the transition state(s) for the conjugation–elimination steps of bound ligands, summarized in Figure 1. To determine the magnitude of these solvent isotope effects or the number of protons in flight in the transition states would require proton inventory studies for both forward and reverse reactions [52,53,54].

In addition to solvent kinetic isotope effects, a primary KIE is also expected in D_2_O for the deuterium exchange reaction to produce ^2^H-NE from GS-NE. Once deuterium is incorporated at C2, a significant primary isotope would be expected to remove it and regenerate NE [52,55]. Thus, the flux of [GST•GS-NE] backwards to [GST•GSH•NE] would be expected to be faster in H_2_O than in our experiments with D_2_O. The magnitude of primary isotope effects for E2 elimination reactions can vary between 2–8 [56]. If the primary isotope effect were dominant over the solvent isotope effects, the measured partition ratio of forward flux to reverse reaction measured by deuterium exchange could be a significant overestimation and the reverse partitioning of the [GST•GS-NE] intermediate could be much greater in H_2_O than observed in our experiments. The magnitude of the primary KIE could, however, be masked (decreased) if the proton removal from C2 in the reverse direction is not stereospecific, and this further complicates any prediction of the expected KIE. The stereochemical course of the conjugation of GSH to the prochiral C3 of NE catalyzed by GSTA4-4 is not defined, in contrast to the reaction with HNE, which occurs at the si face of both (R) and (S) HNE [57].

Finally, secondary deuterium isotope effects on the reverse reaction would also be expected as the rehybridization of C3 from sp3 to sp2 would be faster with deuterium incorporated at C2 [55]. This would yield a faster reverse rate in D_2_O than in H_2_O and have the opposite effect of the primary isotope effect. However, secondary isotope effects are small (<<2) and this would not be likely to increase the apparent rate significantly. In summary, many experiments with various combinations of isotopic solvent and substrates would be required to precisely measure the partition ratio of the forward reaction to GS-NE vs. the reverse reaction to substrates. However, based on the expected directions and magnitudes of solvent and primary kinetic isotope effects, the estimated ratio of forward-to-reverse flux in Table 2 may be an upper limit to the ratio of k_cat,f_/k_ex_ in H_2_O.

Regardless of the complexity of the kinetics and stereochemical course of the reaction, the results provide the first indication that GSTA4-4 is not specifically evolved to optimize the flux of alkenals to their GSH conjugates. This is particularly apparent in comparison to GSTA1-1, which is a very poor catalyst of the Michael addition and the *retro* Michael addition with NE, but has a higher relative commitment to catalysis in the forward direction once GS-NE is formed on the enzyme. This is a striking difference in light of the much greater specificity of GSTA4-4 for long-chain alkenals and the extreme substrate promiscuity of GSTA1-1. In turn, this raises the interesting speculation that GSTA4-4 is not evolutionarily optimized to clear lipid alkenals, despite its high substrate specificity compared to other canonical cytosolic GSTs. Rather GSTA4-4 might have evolved to balance the clearance of lipid alkenals with the regeneration of GSH and alkenal to maintain Nrf2-dependent oxidative stress responses or regenerate GSH during extreme oxidative stress.

## 5. Conclusions

Lipid alkenals play dual roles as electrophilic toxins and ‘second messengers’ that ensure an adequate antioxidant response by activating the Nrf2 transcriptional activator. GSTA4-4 provides a metabolic clearance pathway for lipid alkenals via GSH conjugation, including 2-nonenal (NE). Results presented here with NE reveal that GSTA4-4 efficiently catalyzes the regeneration of GSH and NE from the Michael adduct GS-NE, and the reverse reaction is characterized by remarkably low K_M,GS-NE_ compared to the K_M_ values for GSH or NE in the forward reaction. Furthermore, GSTA4-4 catalyzes the exchange of solvent deuterons from D_2_O specifically at C2 of starting NE on a similar time scale as GS-NE formation. This underscores the high degree of reversibility for GSTA4-4 catalyzed Michael addition. The isotope exchange was dependent on the presence of GSH, indicating that exchange occurs from an intermediate formed en route to GS-NE. The results suggest a possible contribution of GSTA4-4 to the homeostasis during oxidative stress by GSTA4-4, which can readily regenerate GSH or lipid alkenal. Restoring GSH and generating low levels of lipid alkenal could provide short-term and long-term benefits during oxidative stress.

## Figures and Tables

**Table 1:** Kinetic parameters for forward and reverse reactions.

		Forward Reaction				Reverse Reaction	
GSTA Isoform (Assay)	K_M,GSH_ (μM)	K_M,NE_ (μM)	k_cat,f_ (s^−1^)	k_cat,f_/K_M_ (μM^−1^s^−1^)	K_M,GS-NE_ (μM)	k_cat,r_ (s^−1^)	k_cat,r_/K_M,GS-NE_ (μM^−1^s^−1^)
GSTA4-4 (UV)	530 ± 100	NA	89 ± 6	0.17	19 ± 5	1.2 ± 0.1	0.06
GSTA4-4 (LC-MS)	NA	195 ± 49	184 ± 14	0.95	NA	NA	NA
GSTA1-1 (LC-MS)	NA	71 ± 19	0.35 ± 0.1	0.0042	ND	ND	ND

NA, not applicable; the parameters for the reverse reaction were determined only by the UV assay. The K_M_ for NE (K_M,NE_) and GSH (K_M,GSH_) is reported for the LC-MS and UV assay, respectively. ND, not detected. The GSTA1-1 yields insufficient NE to measure under conditions used for GSTA4-4. All experiments were at 25 °C.

**Table 2:** Rate of ^1^H-NE depletion (k_ex_) and GS-NE formation (k_cat,f_) and for GSTA4-4 and GSTA1-1.

GSTA Isoform	H/D Exchange, k_ex_ (s^−1^)	GS-NE Formation, k_cat,f_ (s^−1^)	Ratio k_cat,f_/k_ex_
GSTA4-4	63 ± 17	184 ± 14	2.9
GSTA1-1	0.017 ± 0.18	0.35 ± 0.1	20.5

All reactions were at 25 °C.

## Data Availability

Not applicable.

## Supplementary Materials

The following supporting information can be downloaded at: https://www.mdpi.com/article/10.3390/biom13020329/s1, Figure S1: ^1^H-NMR Spectra for NE, GSH, GSSG, and GS-NE; Figure S2: 2D COSY Spectrum of synthetic GS-NE; Figure S3: 2D TOCSY spectrum of synthetic GS-NE; Figure S4: Full scan (top) and MS-MS spectra of synthetic GS-NE; Figure S5: Demonstration that deuterium (^2^H) is exchanged specifically at C2 of NE; Figure S6: H/D exchange of NE requires GSH.
