# Supplementary material for: Reversibility and Low Commitment to Forward Catalysis in the Conjugation of Lipid Alkenals by Glutathione Transferase A4-4
Source: Biomolecules. 2023 Feb 9;13(2):329. doi: 10.3390/biom13020329 (PMC9953347; doi:10.3390/biom13020329)
Supplement: Supplementary file 1 [file biomolecules-13-00329-s001.zip › biomolecules-2165690-supplementary.pdf]

SUPPORTING INFORMATION

Reversibility and Low Commitment to Forward Catalysis in the Conjugation of Lipid Alkenals by Glutathione Transferase A4-4

Michele Scian † Lorela Paço †, Taylor A. Murphree, Laura M. Shireman and William M. Atkins *

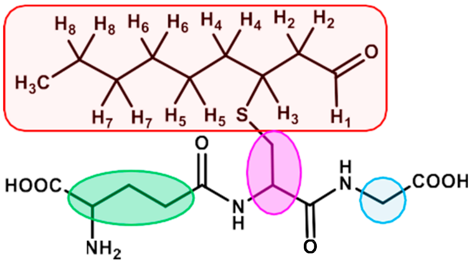

1D NMR.

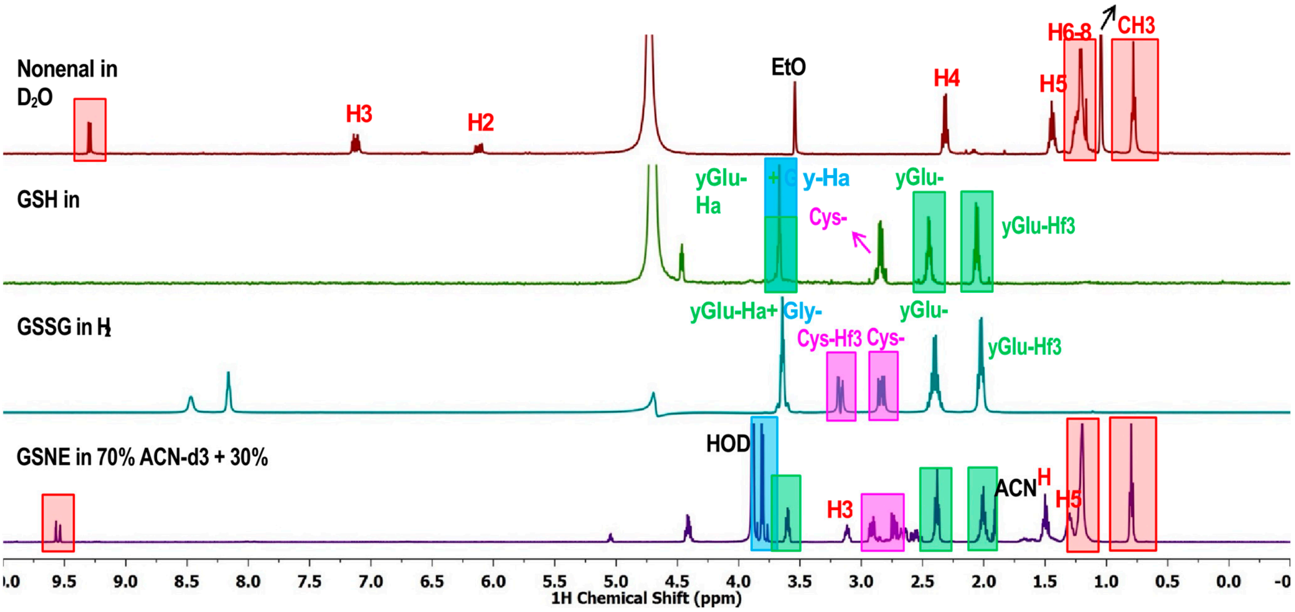

EtOH

Figure S1. 1H-NMR Spectra for NE, GSH, GSSG and GS-NE. Spectra are shown top to bottom and peaks are color coded according to the scheme shown for the molecular structure of GS-NE. The black arrow above the top spectrum at ~1 ppm is from residual EtOH.

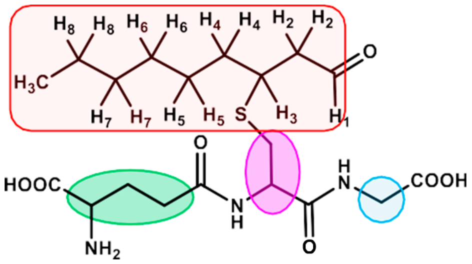

NMR assignment:

2D-COSY.

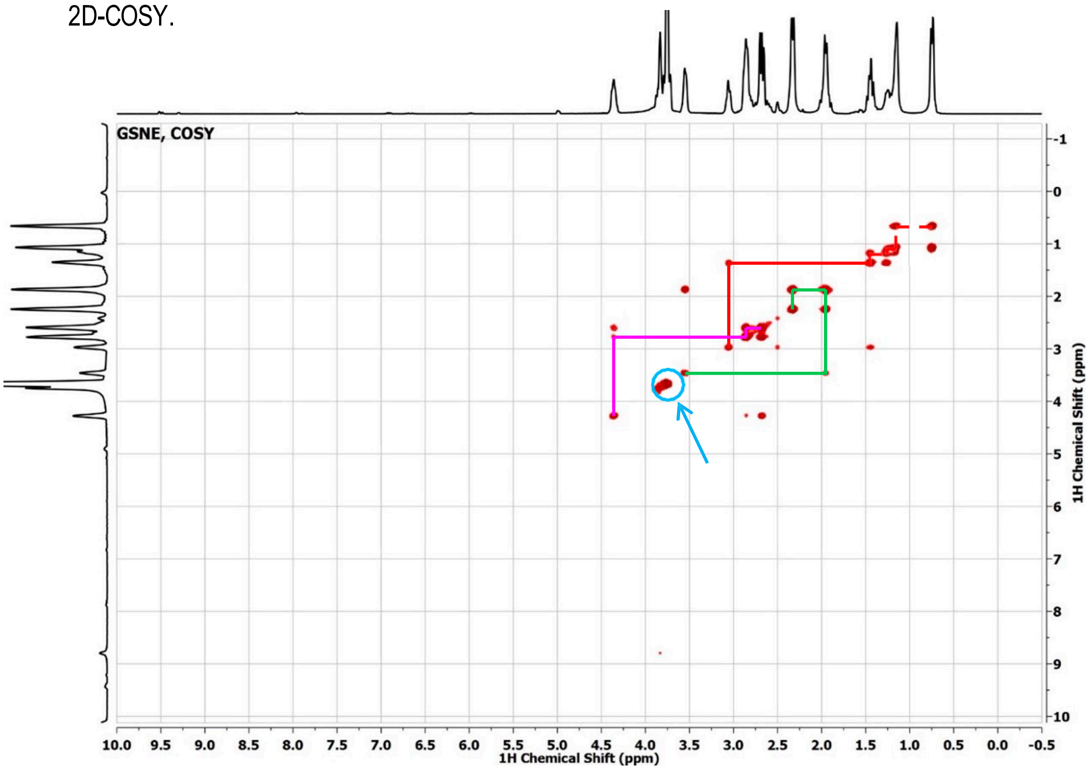

Figure S2. 2D COSY Spectrum of synthetic GS-NE.

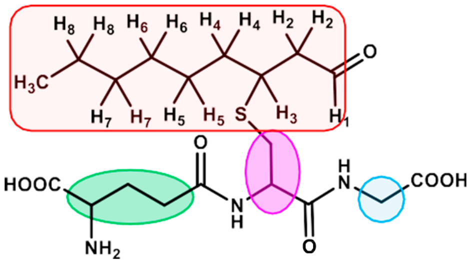

NMR assignment:

2D-TOCSY.

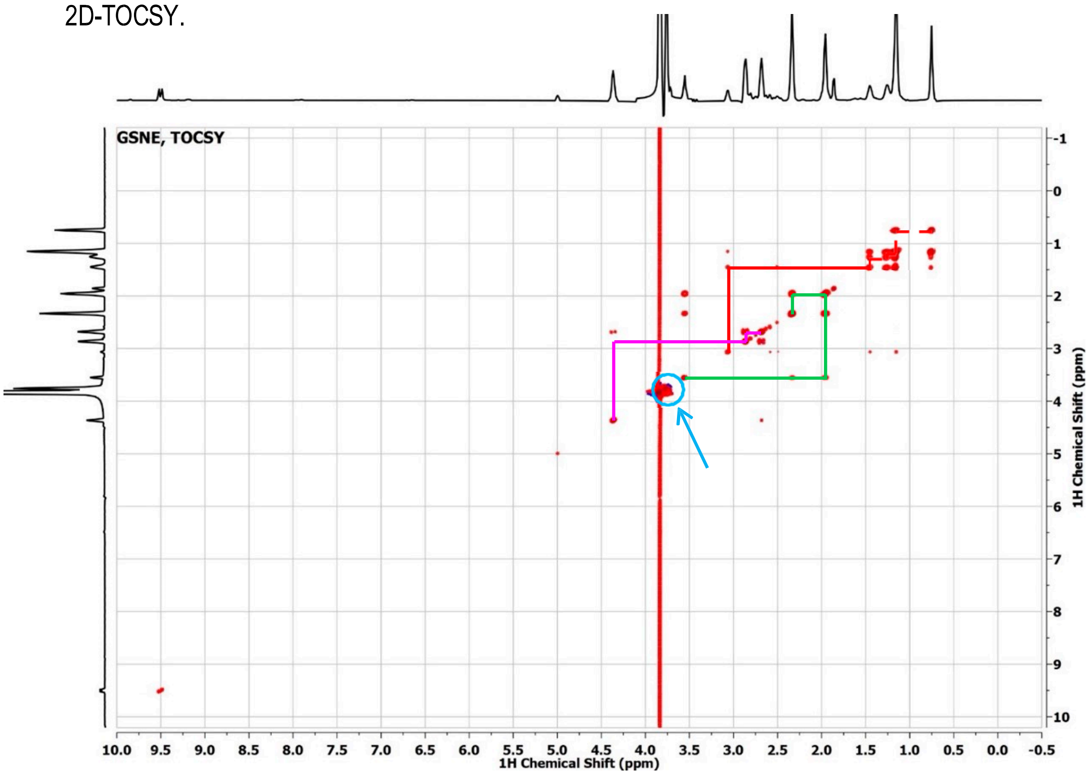

Figure S3. 2D TOCSY spectrum of synthetic GS-NE.

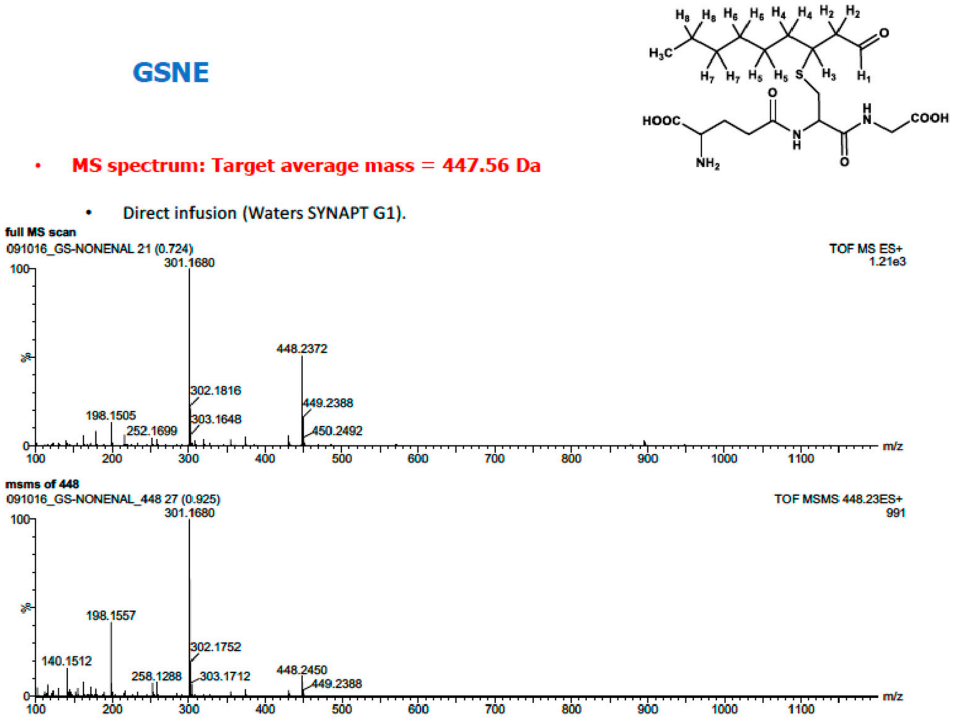

Figure S4. Full scan (top) and MS-MS spectra of synthetic GS-NE.

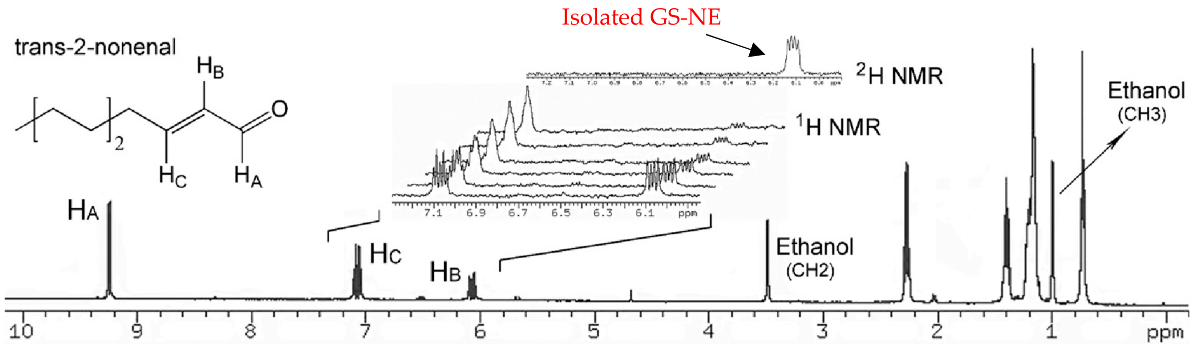

Figure S5. Demonstration that deuterium ($_2$H) is exchanged specifically at C2 of NE. The proton NMR assignment is shown where H_A, H_B, and H_C, correspond to H1, H2, H3, respectively, which show the changes in coupling or intensity described in the main text during incubation in D_2O with GSTA4 and GSH. The NE was isolated and analyzed by $_2$H-NMR. The proton at C2 is specifically converted to a deuteron, $_2$H, as shown in the inset deuterium spectrum above the proton spectrum.

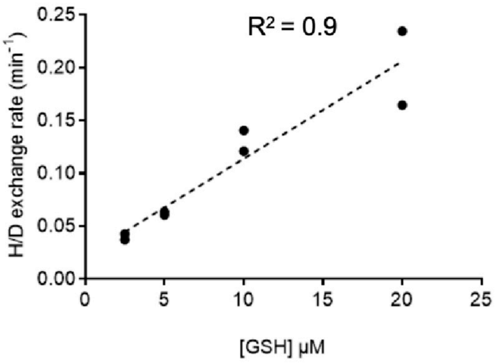

Figure S6. H/D exchange of NE requires GSH. The rate of H/D exchange was determined at 25 C by fitting the integrated peak area for H1 of NE at varying times, for each [GSH] shown. The data are fit to a line for visualization. The concentration range of [GSH] is sufficiently below the $K_{M,GSH}$ that the concentration dependence approximates a line. hGSTA4-4 was 10 nM with 100 mM deuterated NaPi buffer at pH* = 6.66 (purged with Argon for 1 min). To appropriate amounts of these solutions, freshly made L-Glutathione (≥98%, Sigma-Aldrich, St. Louis, MO) solutions were added (reduced GSH in 100 mM deuterated NaPi, pH* = 6.66, purged with Argon for 1 min) to a final enzyme concentration of 0.1 mM and [GSH] = 1.5, 2.5, 5.0, 10 mM (in 600 mL total). Lastly, NE (27.5 mM stock solution in ethanol-d6) to a final concentration 275 mM was added, immediately before starting the NMR experiment. The cosolvent concentration in each of the NMR samples was kept to 1% *v/v*.
